# Supplementary material for: Geospatial Analysis of Individual and Community-Level Socioeconomic Factors Impacting SARS-CoV-2 Prevalence and Outcomes
Source: medRxiv. 2020 Sep 30:2020.09.30.20201830. Preprint. [Version 1] doi: 10.1101/2020.09.30.20201830 (PMC7536884; doi:10.1101/2020.09.30.20201830)
Supplement: Supplement 1 [file media-1.docx]

**SUPPLEMENT - Geospatial Analysis of Individual and Community-Level Socioeconomic Factors Impacting
SARS-CoV-2 Prevalence and Outcomes**

Supplemental Table 1 – Page 2

Supplemental Table 2 – Page 3

Supplemental Table 3 – Page 4-5

Supplemental Table 4 – Page 6-7

Supplemental Table 5 – Page 8

Supplemental Table 6 – Page 9

Supplemental Table 7 – Page 10-13

**Supplemental Figure 1** – Website summarizing key information from this analysis, including interactive choropleth maps depicting SARS-CoV-2 outcomes in the Mass General Brigham hospital system and American Community Survey variables by census tract in Eastern Massachusetts. Available at <https://covidses.github.io>, with code repository at <https://github.com/covidses/analysis_scripts>.

*Supplemental Table 1: American Community Survey (ACS) Tables Used to Define Census Tract-level Sociodemographic Variables*

| Race Assigned | Countries | Languages |
| --- | --- | --- |
| White, non-Hispanic | Albania, Andorra, Australia, Belarus, Bosnia and Herzegovina, Bulgaria, Canada, Czech Republic, France, Germany, Greece, Ireland, Italy, Moldova, Poland, Portugal, Russia, Spain, Sweden, Ukraine, United Kingdom | Albanian, Bulgarian, Croatian, Danish, Greek, Italian, Russian, Swedish |
| Black, non-Hispanic | Angola, Antigua and Barbuda, Barbados, Cameroon, Cayman Islands, Congo, Curacao, Democratic Republic of the Congo, Eritrea, Ethiopia, Ghana, Guadeloupe, Guinea, Guinea-Bissau, Haiti, Kenya, Liberia, Nigeria, Sao Tome and Principe, Sierra Leone, Somalia, South Sudan, Sudan, U.S. Virgin Islands, Uganda | Amharic, Creole, Eritrean, Haitian-Creole, Somali, Swahili |
| Asian, non-Hispanic | Bangladesh, Bhutan, British Indian Ocean Territory, Burma, Cambodia, China, Hong Kong, India, Japan, Macao, Nepal, Palau, Philippines, Sri Lanka, Taiwan, Thailand, Vietnam | Bengali, Cambodian, Chinese - Cantonese, Chinese - Mandarin, Gujarati, Hindi, Japanese, Kannada, Khmer, Laotian, Nepali, Popo, Punjabi, Tagalog, Thai, Urdu, Vietnamese |
| Hispanic | Argentina, Bolivia, Brazil, Chile, Colombia, Costa Rica, Cuba, Dominican Republic, Ecuador, El Salvador, Guatemala, Honduras, Mexico, Nicaragua, Panama, Paraguay, Peru, Puerto Rico, Uruguay, Venezuela | Portuguese, Spanish |

*Supplemental Table 2: Number of individuals with Other or Missing race who were reassigned based on Country of Origin or Language*

| Recorded Race/Ethnicity | Assigned Race/Ethnicity | Total Number Reassigned | Number Reassigned Based on Country of Origin | Number Reassigned Based on Language |
| --- | --- | --- | --- | --- |
| Other | White, non-Hispanic | 34 | 31 | 3 |
|  | Black, non-Hispanic | 47 | 42 | 5 |
|  | Asian, non-Hispanic | 27 | 18 | 9 |
|  | Hispanic | 358 | 224 | 134 |
|  | Other | 649 | n/a | n/a |
| Missing | White, non-Hispanic | 28 | 17 | 11 |
|  | Black, non-Hispanic | 33 | 15 | 18 |
|  | Asian, non-Hispanic | 31 | 10 | 21 |
|  | Hispanic | 301 | 36 | 265 |
|  | Missing | 2855 | n/a | n/a |

*Supplemental Table 3: American Community Survey (ACS) Tables Used to Define Census Tract-level Sociodemographic Variables*

| Variable | ACS Variable Used |
| --- | --- |
| Age       Percent age ≥ 64 | B01001020 - B01001025, B01001044-B01001049 |
| Race/Ethnicity, mean (IQR)       % White, non-Hispanic       % Black, non-Hispanic       % Asian, non-Hispanic       % Hispanic | B03002003  B03002004  B03002006  B03002012 |
| % Foreign-born | B05012003 |
| Insurance       % Private, non-Medicare           % Medicare         % Medicaid, MassHealth         % Safety Net/None | B27010004 - B27010005, B27010011, B27010012, B27010014, B27010020, B27010021, B27010027, B27010028, B27010030, B27010036, B27010037, B27010043, B27010044, B27010045, B27010047, B27010053, B27010054, B27010059 - B27010061, B27010063  B27010006, B27010012, B27010013,B27010022, B27010028, B27010029, B27010038, B27010044, B27010045, B27010046, B27010055, B27010060, B27010061, B27010062  B27010007, B27010013, B27010023, B27010029, B27010039, B27010046, B27010062    B27010066 |
| Population and Population Density       Total Population       Land Area | B01003001  Tiger/Line Shapefiles |
| Income and Income Inequality       Median household income       % public assistance income       GINI Index | B19013001  B19057002  B19083001 |
| Poverty       % living below 100% federal poverty level       % living below 150% federal poverty level       % living below 200% federal poverty level | C17002002 - C17002003  C17002002 - C17002005  C17002002 - C17002007 |
| Education       % without high school diploma       % with college degree | B15003002 - B15003016  B15003022 - B15003025 |
| Employment and Occupation       % unemployed       % essential workers | B23025005  C24010012, C24010016, C24010020, C24010021 - C24010026, C24010030, C24010034, C24010048, C24010052, C24010056, C24010057 - C24010062, C24010066, C24010070 |
| Household Crowding       % with >1 occupant per room       % with >1.5 occupants per room       % with >2 occupants per room | B25014005 - B25014007, B25014011 - B25014013  B25014006 - B25014007, B25014012 - B25014013  B25014007, B25014013 |
| Household Crowding       % households with 2 or more members       % households with 3 or more members       % households with 4 or more members       % households with 5 or more members       % households with 6 or more members       % households with 7 or more members | B11016003 - B11016008, B11016011 - B11016016  B11016004 - B11016008, B11016012 - B11016016  B11016005 - B11016008, B11016013 - B11016016  B11016006 - B11016008, B11016014 - B11016016  B11016007 - B11016008, B11016015 - B11016016  B11016008 - B11016008, B11016016 - B11016016 |
| Housing Factors       Median Home Value       % plumbing facilities for all housing units | B25077001  B25047003 |
| Household Type       % 1 unit in structure       % ≥ 2 units in structure       % ≥ 3 units in structure       % ≥ 5 units in structure       % ≥ 10 units in structure       % ≥ 20 units in structure       % ≥ 50 units in structure | B25032003 - B25032004, B25032014 - B25032015  B25032005, B25032010, B25032016 - B25032021  B25032006, B25032010, B25032017 - B25032021  B25032007, B25032010, B25032018 - B25032021  B25032008, B25032010, B25032019 - B25032021  B25032009, B25032010, B25032020 - B25032021  B25032010, B25032010, B25032021 - B25032021 |
| Transportation       % commute by walk       % commute by public transit       % commute by vehicle       % work from home | B08301019  B08301010  B08301002, B08301017  B08301021 |

*Supplemental Table 4: Distribution Characteristics of Census Tract-level Sociodemographic Variables from the American Community Survey (ACS) among Included Census Tracts*

|  | Min | Max | Mean | Median | 25th %ile | 75th %ile | 95th %ile | 99th %ile |
| --- | --- | --- | --- | --- | --- | --- | --- | --- |
| Percent age ≥ 65 | 0 | 0.6538 | 0.159 | 0.154 | 0.1168 | 0.1891 | 0.2731 | 0.3998 |
| Race/Ethnicity, mean (IQR) |  |  |  |  |  |  |  |  |
| % White, non-Hispanic | 0.006 | 0.9989 | 0.7142 | 0.7995 | 0.5879 | 0.8972 | 0.9605 | 0.9804 |
| % Black, non-Hispanic | 0 | 0.8186 | 0.0708 | 0.0276 | 0.0092 | 0.0718 | 0.3452 | 0.6343 |
| % Asian, non-Hispanic | 0 | 0.5615 | 0.0645 | 0.0346 | 0.0104 | 0.087 | 0.2258 | 0.3896 |
| % Hispanic | 0 | 0.971 | 0.119 | 0.0563 | 0.0257 | 0.1401 | 0.459 | 0.7899 |
| % Foreign-born | 0.0097 | 0.6483 | 0.171 | 0.1378 | 0.068 | 0.2469 | 0.4249 | 0.5171 |
| Insurance |  |  |  |  |  |  |  |  |
| % Private, non-Medicare | 0 | 0.9696 | 0.6647 | 0.7111 | 0.5712 | 0.8008 | 0.8802 | 0.9125 |
| % Medicare | 0 | 0.4197 | 0.1351 | 0.1298 | 0.104 | 0.1574 | 0.2233 | 0.3146 |
| % Medicaid, MassHealth | 0 | 0.7862 | 0.1962 | 0.1407 | 0.0784 | 0.2642 | 0.5449 | 0.6469 |
| % Safety Net/None | 0 | 0.0236 | 0.0007 | 0 | 0 | 0 | 0.004 | 0.0102 |
| % Safety Net/None, dichotomized | 0 | 1 | 0.1486 | 0 | 0 | 0 | 1 | 1 |
| Population and Population Density |  |  |  |  |  |  |  |  |
| Log (Total Population) | 1.4314 | 4.1158 | 3.6437 | 3.6667 | 3.5389 | 3.7719 | 3.8939 | 3.9501 |
| Log (Population Density) | 0.9813 | 4.5815 | 3.0248 | 3.0534 | 2.5189 | 3.5576 | 4.065 | 4.3117 |
| Income and Income Inequality |  |  |  |  |  |  |  |  |
| Median household income | 2.499 | 250.001 | 83.8301 | 79.8555 | 56.9192 | 104.8335 | 153.2397 | 184.4047 |
| % public assistance income | 0 | 0.25 | 0.028 | 0.0182 | 0.0088 | 0.0363 | 0.089 | 0.1357 |
| GINI Index | 0.0844 | 0.8123 | 0.4346 | 0.427 | 0.3938 | 0.4678 | 0.5485 | 0.6037 |
| Poverty |  |  |  |  |  |  |  |  |
| % living below 100% federal poverty level (FPL) | 0 | 0.8062 | 0.1141 | 0.0777 | 0.0423 | 0.1558 | 0.3203 | 0.4887 |
| % living below 150% FPL | 0 | 0.8722 | 0.1779 | 0.1282 | 0.0756 | 0.2446 | 0.463 | 0.638 |
| % living below 200% FPL | 0 | 0.978 | 0.2412 | 0.1853 | 0.1166 | 0.3348 | 0.577 | 0.7169 |
| Education |  |  |  |  |  |  |  |  |
| % without high school diploma | 0 | 0.6749 | 0.1013 | 0.0651 | 0.0355 | 0.1392 | 0.3019 | 0.4181 |
| % with college degree | 0 | 1 | 0.4284 | 0.4011 | 0.2575 | 0.5835 | 0.8178 | 0.8901 |
| Employment and Occupation |  |  |  |  |  |  |  |  |
| % unemployed | 0 | 0.2381 | 0.0371 | 0.0329 | 0.0228 | 0.0464 | 0.0763 | 0.1225 |
| % essential workers | 0 | 1 | 0.4539 | 0.4525 | 0.3458 | 0.5581 | 0.6959 | 0.7874 |
| Household Crowding |  |  |  |  |  |  |  |  |
| % with >1 occupant per room | 0 | 0.2076 | 0.021 | 0.0125 | 0 | 0.0287 | 0.0731 | 0.1363 |
| % with >1.5 occupants per room | 0 | 0.1282 | 0.0075 | 0 | 0 | 0.0093 | 0.0341 | 0.0602 |
| % with >2 occupants per room | 0 | 0.0975 | 0.0021 | 0 | 0 | 0 | 0.0135 | 0.0339 |
| % with >2 occupants per room, dichotomized | 0 | 1 | 0.1696 | 0 | 0 | 0 | 1 | 1 |
| Household Occupancy |  |  |  |  |  |  |  |  |
| % households with ≥ 2 members | 0 | 1 | 0.7104 | 0.7256 | 0.6508 | 0.7854 | 0.8661 | 0.9035 |
| % households with ≥ 3 members | 0 | 0.8051 | 0.383 | 0.3888 | 0.3148 | 0.4615 | 0.5504 | 0.6203 |
| % households with ≥ 4 members | 0 | 0.5586 | 0.2178 | 0.2151 | 0.1586 | 0.2785 | 0.36 | 0.4215 |
| % households with ≥ 5 members | 0 | 0.3316 | 0.0806 | 0.0751 | 0.0487 | 0.1079 | 0.1642 | 0.2158 |
| % households with ≥ 6 members | 0 | 0.1756 | 0.0265 | 0.0213 | 0.0092 | 0.0374 | 0.0741 | 0.1051 |
| % households with ≥ 7 members | 0 | 0.0808 | 0.0088 | 0.0042 | 0 | 0.0123 | 0.0363 | 0.0563 |
| Housing Factors |  |  |  |  |  |  |  |  |
| Median Home Value | 111.5 | 2000.001 | 417.211 | 366.35 | 270.4 | 492.075 | 852.5 | 1161.21 |
| % housing units lacking plumbing facilities | 0 | 1 | 0.0131 | 0.0034 | 0 | 0.0155 | 0.0401 | 0.0857 |
| Housing Type |  |  |  |  |  |  |  |  |
| % 1 unit in structure | 0 | 1 | 0.5568 | 0.6178 | 0.2721 | 0.8304 | 0.9567 | 0.9889 |
| % ≥ 2 units in structure | 0 | 1 | 0.38 | 0.3247 | 0.1315 | 0.6223 | 0.8564 | 0.9577 |
| % ≥ 3 units in structure | 0 | 1 | 0.3026 | 0.2291 | 0.0946 | 0.4736 | 0.8108 | 0.9468 |
| % ≥ 5 units in structure | 0 | 0.9822 | 0.1968 | 0.1346 | 0.0509 | 0.2725 | 0.6336 | 0.9041 |
| % ≥ 10 units in structure | 0 | 0.9796 | 0.1441 | 0.0859 | 0.0252 | 0.1949 | 0.5151 | 0.8435 |
| % ≥ 20 units in structure | 0 | 0.9869 | 0.1095 | 0.0542 | 0.011 | 0.1393 | 0.4516 | 0.7129 |
| % ≥ 50 units in structure | 0 | 0.9543 | 0.0653 | 0.0172 | 0 | 0.075 | 0.2985 | 0.5713 |
| Transportation |  |  |  |  |  |  |  |  |
| % commute by walk | 0 | 0.7859 | 0.0556 | 0.0219 | 0.007 | 0.054 | 0.2688 | 0.5248 |
| % commute by public transit | 0 | 0.7187 | 0.1075 | 0.051 | 0.0163 | 0.1541 | 0.3946 | 0.5028 |
| % commute by vehicle | 0 | 1 | 0.7647 | 0.8439 | 0.7018 | 0.9016 | 0.9513 | 0.9757 |
| % work from home | 0 | 0.7692 | 0.0514 | 0.0433 | 0.0241 | 0.0674 | 0.1207 | 0.1757 |

*Supplemental Table 5: Base Models for hospitalization and death, excluding individual race or language*

|  | SARS-CoV-2-related Hospitalization | | SARS-CoV-2-related Mortality | |
| --- | --- | --- | --- | --- |
|  | Base Model without Race | Base Model without Language | Base Model without Race | Base Model without Language |
| Number of individuals in analysis, n | 9,839 | | 3,009 | |
| **Individual Characteristics** |  | |  | |
| Age | 1.04 (1.04, 1.04) | 1.04 (1.04, 1.04) | 1.06 (1.05, 1.07) | 1.06 (1.05, 1.07) |
| Sex |  |  |  |  |
| Female | Ref | Ref | Ref | Ref |
| Male | 1.51 (1.36, 1.66) | 1.49 (1.35, 1.65) | 1.65 (1.33, 2.05) | 1.65 (1.33, 2.05) |
| Race/Ethnicity |  |  |  |  |
| White, non-Hispanic |  | Ref |  | Ref |
| Black, non-Hispanic |  | 1.49 (1.25, 1.77) |  | 0.86 (0.62, 1.28) |
| Asian, non-Hispanic |  | 1.68 (1.27, 2.23) |  | 0.70 (0.39, 1.27) |
| Hispanic |  | 0.97 (0.84, 1.12) |  | 0.94 (0.68, 1.28) |
| Other |  | 1.58 (1.00, 2.50) |  | 0.47 (0.15, 1.44) |
| Missing |  | 0.45 (0.34, 0.58) |  | 1.07 (0.59, 1.94) |
| Language, n(%) |  |  |  |  |
| English | Ref |  | Ref |  |
| Spanish | 0.98 (0.86, 1.12) |  | 0.88 (0.64, 1.21) |  |
| Other | 1.42 (1.16, 1.74) |  | 1.07 (0.76, 1.49) |  |
| Missing | 0.32 (0.23, 0.46) |  | 1.13 (0.50, 2.59) |  |
| Insurance, n(%) |  |  |  |  |
| Private, non-Medicare | Ref | Ref | Ref | Ref |
| Medicare | 2.17 (1.89, 2.50) | 2.20 (1.92, 2.54) | 1.90 (1.43, 2.52) | 1.87 (1.41, 2.49) |
| Medicaid, MassHealth | 1.56 (1.36, 1.79) | 1.62 (1.42, 1.86) | 1.04 (0.67, 1.62) | 1.04 (0.67, 1.60) |
| Safety Net/Unlisted | 0.35 (0.27, 0.46) | 0.37 (0.29, 0.48) | 0.74 (0.22, 2.49) | 0.68 (0.20, 2.29) |
| **Census Tract Characteristics** |  | |  | |
| Per Capita Testing Rate | **0.00 (0.00, 0.40)** | **0.00 (0.00, 0.30)** | 0.27 (0.00, 192.42) | 0.26 (0.00, 195.69) |

*Supplemental Table 6: Baseline Characteristics of Individuals Included in the Analysis for Each Outcome: Sensitivity Analysis in which those with missing, but not Other, race were reassigned race based on country of origin and language preference.*

|  | Total Tested^a^ | Any Positive Test^b^ | Hospitalized, given positive test^b^ | Deceased, given hospitalization^b^ |
| --- | --- | --- | --- | --- |
|  | (n=57,865) | (n=9,839, 17.0%) | (n=3,009, 30.6%) | (n=524, 17.4%) |
| Age, mean (SD) | 52.32 (19.31) | 51.23 (19.89) | 62.72 (18.90) | 77.05 (13.42) |
| Age Groups, n (%) |  |  |  |  |
| < 50 years | 25,877 (44.72) | 4,827 (18.65) | 752 (15.58) | 19 (2.53) |
| 50-64 years | 15,041 (25.99) | 2,475 (16.46) | 784 (31.68) | 73 (9.31) |
| 65-79 years | 11,837 (20.46) | 1,479 (12.49) | 813 (54.97) | 176 (21.65) |
| ≥ 80 years | 5,110 (8.83) | 1,058 (20.70) | 660 (62.38) | 256 (38.79) |
| Sex, n (%) |  |  |  |  |
| Female | 32,260 (55.75) | 4,966 (15.39) | 1,396 (28.11) | 224 (16.05) |
| Male | 25,586 (44.22) | 4,871 (19.04) | 1,613 (33.11) | 300 (18.60) |
| Race/Ethnicity, n (%)^c^ |  |  |  |  |
| White, non-Hispanic | 34,364 (59.39) | 3,229 (9.40) | 1,304 (40.38) | 320 (24.54) |
| Black, non-Hispanic | 5,481 (9.47) | 1,218 (22.22) | 462 (37.93) | 70 (15.15) |
| Asian, non-Hispanic | 2,050 (3.54) | 293 (14.29) | 116 (39.59) | 15 (12.93) |
| Hispanic | 12,000 (20.74) | 4,126 (34.38) | 958 (23.22) | 89 (9.29) |
| Other | 1,115 (1.93) | 275 (24.66) | 78 (28.36) | 12 (15.38) |
| Missing | 2,855 (4.93) | 698 (24.45) | 91 (13.04) | 18 (19.78) |
| Language, n(%) |  |  |  |  |
| English | 46,086 (79.64) | 5,403 (11.72) | 1,855 (34.33) | 369 (19.89) |
| Spanish | 7,602 (13.14) | 3,289 (43.26) | 824 (25.05) | 77 (9.34) |
| Other | 2,118 (3.66) | 591 (27.90) | 279 (47.21) | 69 (24.73) |
| Missing | 2,059 (3.56) | 556 (27.00) | 51 (9.17) | 9 (17.65) |
| Insurance, n(%) |  |  |  |  |
| Private, non-Medicare | 29,740 (51.40) | 4,031 (13.55) | 935 (23.20) | 85 (9.09) |
| Medicare | 16,480 (28.48) | 2,311 (14.02) | 1,365 (59.07) | 400 (29.30) |
| Medicaid, MassHealth | 8,397 (14.51) | 2,435 (29.00) | 630 (25.87) | 36 (5.71) |
| Safety Net/Unlisted | 3,248 (5.61) | 1,062 (32.70) | 79 (7.44) | 3 (3.80) |

^a^ Percentages represent the percent of individuals tested who fell into a category. (e.g. 44.72% of all individuals tested were male).
^b^ Percentages represent the percent of individuals experiencing that outcome, among all eligible to experience the outcome in that cell (e.g. 19.04% of men tested for SARS-CoV-2 received a positive test result).
^c^ Includes reassigned race (based on country of origin or language) for those with recorded missing race.

*Supplemental Table 7: Individual and Census Tract Correlates of Individual SARS-CoV-2 Test Positivity, SARS-CoV-2 Related Admission, and SARS-CoV-2-related Death in Base (Individual Characteristics only), Base-Plus (Base Model Covariates plus Single Census Tract-Level Variables), and Final Multivariable Models: Sensitivity Analysis in which those with missing, but not Other, race were reassigned race based on country of origin and language preference.*

|  | SARS-CoV-2 Infection | | | SARS-CoV-2-related Hospitalization | | | SARS-CoV-2-related Mortality | | |
| --- | --- | --- | --- | --- | --- | --- | --- | --- | --- |
|  | Base Model^a^ | Base-Plus Models^b^ | Full Model^c^ | Base Model^a^ | Base-Plus Models^b^ | Full Model^c^ | Base Model^a^ | Base-Plus Models^b^ | Full Model^c^ |
| Number of individuals in analysis, n | 57,865 | | | 9,839 | | | 3,009 | | |
| **Individual Characteristics** |  | | |  | | |  | | |
| Age | **1.01 (1.01, 1.01)** |  | **1.01 (1.01, 1.01)** | **1.04 (1.04, 1.04)** |  | **1.04 (1.04, 1.04)** | **1.06 (1.05, 1.07)** |  | **1.06 (1.05, 1.07)** |
| Sex |  |  |  |  |  |  |  |  |  |
| Female | Ref |  | Ref | Ref |  | Ref | Ref |  | Ref |
| Male | **1.30 (1.24, 1.36)** |  | **1.30 (1.24, 1.37)** | **1.50 (1.36, 1.66)** |  | **1.51 (1.36, 1.66)** | **1.64 (1.33, 2.04)** |  | **1.64 (1.33, 2.04)** |
| Race/Ethnicity^d^ |  |  |  |  |  |  |  |  |  |
| White, non-Hispanic | Ref |  | Ref | Ref |  | Ref | Ref |  | Ref |
| Black, non-Hispanic | **2.69 (2.47, 2.92)** |  | **2.60 (2.39, 2.83)** | **1.48 (1.24, 1.76)** |  | **1.40 (1.17, 1.67)** | 1.13 (0.66, 1.94) |  | 0.80 (0.56, 1.13) |
| Asian, non-Hispanic | **1.38 (1.20, 1.58)** | Base | **1.36 (1.18, 1.57)** | **1.56 (1.16, 2.10)** | Base | **1.52 (1.13, 2.05)** | 0.91 (0.44, 1.87) | Base | 0.56 (0.29, 1.09) |
| Hispanic | **2.25 (2.06, 2.45)** | Model | **2.19 (2.01, 2.39)** | 0.86 (0.71, 1.05) | Model | 0.83 (0.68, 1.01) | 0.56 (0.29, 1.09) | Model | 1.13 (0.66, 1.94) |
| Other | **2.09 (1.80, 2.44)** | Plus | **2.06 (1.76, 2.40)** | 1.02 (0.74, 1.41) | Plus | 0.99 (0.72, 1.36) | 1.05 (0.55, 2.01) | Plus | 0.91 (0.44, 1.87) |
| Missing | **2.17 (1.89, 2.49)** | Single | **2.14 (1.86, 2.46)** | **0.70 (0.50, 0.97)** | Single | **0.68 (0.49, 0.95)** | 0.00 (0.00, 0.00) | Single | 1.05 (0.55, 2.01) |
| Language, n(%) |  | Census |  |  | Census |  |  | Census |  |
| English | Ref | Tract-Level | Ref | Ref | Tract-Level | Ref | Ref | Tract-Level | Ref |
| Spanish | **2.38 (2.19, 2.60)** | Variables | **2.36 (2.17, 2.57)** | 1.19 (0.99, 1.44) | Variables | 1.18 (0.98, 1.42) | 1.13 (0.46, 2.75) | Variables | 0.75 (0.43, 1.31) |
| Other | **1.93 (1.73, 2.16)** | Below | **1.90 (1.71, 2.13)** | 1.22 (0.99, 1.51) | Below | 1.19 (0.96, 1.47) | 0.75 (0.43, 1.31) | Below | 1.26 (0.86, 1.84) |
| Missing | **1.64 (1.40, 1.92)** |  | **1.63 (1.40, 1.91)** | **0.43 (0.29, 0.65)** |  | **0.43 (0.29, 0.64)** | 0.71 (0.21, 2.39) |  | 1.13 (0.46, 2.75) |
| Insurance, n(%) |  |  |  |  |  |  |  |  |  |
| Private, non-Medicare | Ref |  | Ref | Ref |  | Ref | Ref |  | Ref |
| Medicare | 0.95 (0.88, 1.02) |  | 0.94 (0.88, 1.01) | **2.22 (1.93, 2.55)** |  | **2.20 (1.91, 2.53)** | **0.43 (0.00, 313.98)** |  | **1.87 (1.40, 2.48)** |
| Medicaid, MassHealth | **1.21 (1.13, 1.30)** |  | **1.20 (1.13, 1.29)** | **1.59 (1.39, 1.82)** |  | **1.58 (1.37, 1.81)** | 1.87 (1.40, 2.48) |  | 1.01 (0.65, 1.57) |
| Safety Net/Unlisted | **1.25 (1.14, 1.37)** |  | **1.24 (1.13, 1.37)** | **0.36 (0.28, 0.47)** |  | **0.36 (0.28, 0.47)** | 1.01 (0.65, 1.57) |  | 0.71 (0.21, 2.39) |
| **Census Tract Characteristics** |  | | |  | | |  | | |
| Per Capita Testing Rate | **2261.71 (259.08, 19743.90)** |  | **1282.86 (125.26, 13137.99)** | **0.00 (0.00, 0.27)** |  | **0.00 (0.00, 0.03)** | 0.43 (0.00, 313.98) |  | 0.43 (0.00, 313.98) |
| Percent age ≥ 65 |  | 0.78 (0.42, 1.43) |  |  | 0.63 (0.18, 2.25) |  |  | 0.79 (0.11, 5.55) |  |
| Race/Ethnicity, mean (IQR) |  |  |  |  |  |  |  |  |  |
| % White, non-Hispanic |  | **0.60 (0.49, 0.74)** |  |  | 0.74 (0.48, 1.13) |  |  | 0.55 (0.30, 0.99) |  |
| % Black, non-Hispanic |  | **2.23 (1.52, 3.26)** | **1.55 (0.97, 2.48)** |  | 0.67 (0.32, 1.42) |  |  | 1.55 (0.60, 3.98) |  |
| % Asian, non-Hispanic |  | 0.95 (0.57, 1.57) | 1.11 (0.69, 1.80) |  | 1.19 (0.42, 3.37) |  |  | 1.43 (0.25, 8.11) |  |
| % Hispanic |  | **1.68 (1.22, 2.30)** | 1.30 (0.83, 2.04) |  | 2.26 (1.22, 4.19) |  |  | 2.00 (0.80, 5.03) |  |
| % Foreign-born |  | **1.99 (1.41, 2.82)** | 0.80 (0.36, 1.77) |  | **3.42 (1.71, 6.83)** | 1.41 (0.58, 3.44) |  | 1.46 (0.50, 4.27) |  |
| Insurance |  |  |  |  |  |  |  |  |  |
| % Private, non-Medicare |  | **0.66 (0.51, 0.85)** | 0.80 (0.36, 1.77) |  | 0.53 (0.32, 0.89) |  |  | 0.51 (0.23, 1.14) |  |
| % Medicare |  | 0.68 (0.34, 1.38) |  |  | 0.97 (0.22, 4.21) |  |  | 0.56 (0.05, 6.11) |  |
| % Medicaid, MassHealth |  | **1.51 (1.14, 1.99)** | 0.50 (0.22, 1.16) |  | 1.56 (0.89, 2.74) |  |  | 1.76 (0.72, 4.30) |  |
| % Safety Net/None |  | 35.17 (0.00, 2.31E7) |  |  | 886.51 (0.00, 2.19E15) |  |  | 1.14E9 (0.00, 1.81E30) |  |
| % Safety Net/None, dichotomized |  | 0.99 (0.92, 1.06) |  |  | 1.01 (0.88, 1.17) |  |  | 1.10 (0.82, 1.47) |  |
| Population and Population Density |  |  |  |  |  |  |  |  |  |
| Log (Total Population) |  | 0.90 (0.74, 1.09) |  |  | 0.90 (0.60, 1.34) |  |  | 1.74 (0.81, 3.73) |  |
| Log (Population Density) |  | **1.25 (1.14, 1.36)** | **1.14 (1.03, 1.27)** |  | **1.25 (1.04, 1.51)** | **1.00 (0.80, 1.24)** |  | 1.15 (0.87, 1.51) |  |
| Income and Income Inequality |  |  |  |  |  |  |  |  |  |
| Median household income (in $1,000s) |  | 0.43 (0.14, 1.34) |  |  | **0.04 (0.00, 0.40)** | 1.37 (0.02, 114.70) |  | 0.05 (0.00, 1.30) |  |
| % public assistance income |  | 2.08 (0.68, 6.37) |  |  | 0.34 (0.03, 3.34) |  |  | 1.35 (0.01, 147.33) |  |
| GINI Index |  | 0.97 (0.56, 1.67) |  |  | 0.57 (0.18, 1.82) |  |  | 1.24 (0.16, 9.52) |  |
| Poverty |  |  |  |  |  |  |  |  |  |
| % living below 100% federal poverty level |  | 1.13 (0.76, 1.70) |  |  | 1.69 (0.74, 3.85) |  |  | 1.61 (0.41, 6.33) |  |
| % living below 150% federal poverty level |  | 1.41 (1.04, 1.92) |  |  | 1.76 (0.94, 3.30) |  |  | 1.82 (0.64, 5.15) |  |
| % living below 200% federal poverty level |  | 1.30 (0.99, 1.70) |  |  | **2.06 (1.20, 3.54)** | 0.65 (0.27, 1.57) |  | 1.86 (0.77, 4.51) |  |
| Education |  |  |  |  |  |  |  |  |  |
| % without high school diploma |  | **2.65 (1.69, 4.14)** | 1.37 (0.66, 2.87) |  | **6.19 (2.51, 15.26)** | **5.24 (1.16, 23.71)** |  | 1.89 (0.43, 8.26) |  |
| % with college degree |  | **0.60 (0.47, 0.76)** | **0.62 (0.39, 0.98)** |  | **0.53 (0.33, 0.84)** | 0.84 (0.37, 1.94) |  | 0.59 (0.31, 1.12) |  |
| Employment and Occupation |  |  |  |  |  |  |  |  |  |
| % unemployed |  | **7.61 (1.79, 32.42)** | 2.79 (0.58, 13.36) |  | 10.51 (0.51, 218.45) |  |  | 2.71 (0.01, 884.98) |  |
| % essential workers |  | **1.57 (1.15, 2.13)** | 0.69 (0.43, 1.13) |  | **2.18 (1.19, 4.01)** | 0.00 (0.00, 0.00) |  | 1.47 (0.60, 3.62) |  |
| Household Crowding |  |  |  |  |  |  |  |  |  |
| % with >1 occupant per room |  | 2.97 (1.08, 8.14) |  |  | 8.79 (1.17, 66.24) |  |  | 13.65 (0.28, 671.13) |  |
| % with >1.5 occupants per room |  | 2.94 (0.32, 27.07) |  |  | 6.75 (0.08, 566.16) |  |  | 231.58 (0.02, 2.30E6) |  |
| % with >2 occupants per room |  | 2.54 (0.07, 85.93) |  |  | 11720.38 (8.54, 1.61E7) |  |  | 0.17 (0.00, 2.94E6) |  |
| % with >2 occupants per room, dichotomized |  | 1.00 (0.94, 1.07) |  |  | **1.16 (1.03, 1.32)** | **1.14 (1.01, 1.30)** |  | 0.96 (0.73, 1.24) |  |
| Household Occupancy |  |  |  |  |  |  |  |  |  |
| % households with ≥ 2 members |  | 1.24 (0.90, 1.71) |  |  | 0.73 (0.38, 1.40) |  |  | 0.85 (0.28, 2.59) |  |
| % households with ≥ 3 members |  | **1.54 (1.15, 2.05)** |  |  | 0.94 (0.52, 1.68) |  |  | 0.83 (0.29, 2.39) |  |
| % households with ≥ 4 members |  | **1.73 (1.20, 2.48)** |  |  | 0.69 (0.33, 1.44) |  |  | 0.88 (0.23, 3.37) |  |
| % households with ≥ 5 members |  | **3.15 (1.73, 5.74)** | **2.20 (1.13, 4.31)** |  | 0.91 (0.27, 3.03) |  |  | 1.82 (0.18, 18.68) |  |
| % households with ≥ 6 members |  | 3.40 (1.13, 10.22) |  |  | 12.46 (1.44, 107.75) |  |  | 11.23 (0.18, 703.60) |  |
| % households with ≥ 7 members |  | 5.28 (0.74, 37.50) |  |  | 30.67 (0.58, 1619.47) |  |  | 10.45 (0.00, 29289.42) |  |
| Housing Factors |  |  |  |  |  |  |  |  |  |
| Median Home Value (in $1,000s) |  | 0.88 (0.71, 1.10) |  |  | 0.64 (0.41, 0.98) |  |  | 0.50 (0.28, 0.89) |  |
| % housing units lacking plumbing facilities |  | 2.64 (0.91, 7.64) |  |  | 9.04 (0.90, 90.43) |  |  | 0.63 (0.01, 50.90) |  |
| Housing Type |  |  |  |  |  |  |  |  |  |
| % 1 unit in structure |  | 0.91 (0.76, 1.10) |  |  | **0.52 (0.36, 0.74)** |  |  | 0.78 (0.48, 1.28) |  |
| % ≥ 2 units in structure |  | 1.12 (0.93, 1.36) |  |  | **2.07 (1.42, 3.02)** | **1.86 (1.03, 3.36)** |  | 1.31 (0.77, 2.25) |  |
| % ≥ 3 units in structure |  | 1.00 (0.82, 1.21) |  |  | 1.43 (0.98, 2.11) |  |  | 1.33 (0.77, 2.31) |  |
| % ≥ 5 units in structure |  | 0.93 (0.77, 1.11) |  |  | 0.89 (0.62, 1.30) |  |  | 0.93 (0.50, 1.72) |  |
| % ≥ 10 units in structure |  | 0.94 (0.78, 1.14) |  |  | 0.95 (0.65, 1.40) |  |  | 1.08 (0.56, 2.09) |  |
| % ≥ 20 units in structure |  | 0.90 (0.74, 1.09) |  |  | 0.83 (0.56, 1.23) |  |  | 0.96 (0.48, 1.93) |  |
| % ≥ 50 units in structure |  | 0.85 (0.68, 1.06) |  |  | 0.81 (0.51, 1.28) |  |  | 0.85 (0.38, 1.94) |  |
| Transportation |  |  |  |  |  |  |  |  |  |
| % commute by walk |  | 1.07 (0.61, 1.89) |  |  | 1.42 (0.46, 4.40) |  |  | 0.77 (0.18, 3.32) |  |
| % commute by public transit |  | 1.09 (0.73, 1.62) |  |  | 1.98 (0.92, 4.27) |  |  | 1.68 (0.62, 4.59) |  |
| % commute by vehicle |  | 1.09 (0.78, 1.53) |  |  | 0.53 (0.28, 1.02) |  |  | 0.93 (0.43, 2.00) |  |
| % work from home |  | **0.26 (0.09, 0.75)** | 0.60 (0.19, 1.91) |  | 1.08 (0.14, 8.36) |  |  | 0.04 (0.00, 1.44) |  |

^a^ The base model is a logistic mixed model including all individual-level characteristics and the census tract-level per capita testing rate in the Mass General Brigham (MGB) hospital system. Bolded values have a false discovery rate (FDR)-adjusted p-value < 0.05.
^b^ The base-plus models are a series of logistic mixed models, each including the covariates of the base model with the addition of a single census tract-level variable as a means of examining the effect of that variable in the absence of other, often correlated, census tract-level covariates. Bolded values have an FDR-adjustment of p-values at a level of 0.05.
^c^ The full multivariable model is a logistic mixed model including the covariates of the base model with the addition of all census tract-level variables which met FDR-adjusted significance thresholds in the base-plus models. If several variables within a highly related group (e.g. percent of households with ≥ 5 members, percent of household with ≥ 6 members, etc.) each met the FDR-adjusted significance threshold in its respective base-plus model, the variable with the most significant p-value was selected for inclusion in the full, multivariable model.
^d^ Includes reassigned race (based on country of origin or language) for those with recorded missing race.
